# Expressions of mitochondria-related genes in pregnant women with subclinical hypothyroidism, and expressions of miRNAs in maternal and cord blood

**DOI:** 10.1186/s13044-023-00180-6

**Published:** 2023-09-18

**Authors:** Julie Kristine Guldberg Stryhn, Jacob Larsen, Palle Lyngsie Pedersen, Peter Haulund Gæde

**Affiliations:** 1 Department of Gynecology and Obstetrics, Slagelse Hospital, Fælledvej 13, 4200 Slagelse, Denmark; 2 Mitochondria Research Unit, Naestved Hospital, Ringstedgade 61, 4700 Naestved, Denmark; 3 Faculty of Health Sciences, University of Southern Denmark, Winsløws Parken, J. B. Winsløws Vej 19, 3, 5000 Odense, Denmark; 4 Department of Clinical Pathology, Roskilde Hospital, Sygehusvej 9, 4000 Roskilde, Denmark; 5 Department of Clinical Biochemistry, Naestved Hospital, Ringstedgade 61, 4700 Naestved, Denmark; 6 Department of Internal Medicine (Endocrinology), Slagelse Hospital, Fælledvej 7, 4200 Slagelse, Denmark

**Keywords:** Subclinical hypothyroidism, Thyroid reference range, Genes, Mitochondrial, MicroRNAs, Pregnancy, Thyrotropin, Blood, Umbilical cord, Biomarker

## Abstract

**Background:**

Subclinical hypothyroidism in pregnancy and definition by upper thyrotropin (TSH) cutoff are controversial. As mitochondria are influenced by thyroid hormones, the purpose in this study was to measure expression of mitochondria-related genes in euthyroid and subclinical hypothyroid pregnant women to obtain more knowledge of potential metabolic consequences of maternal subclinical hypothyroidism. In addition, we wished to test if applied TSH-cutoff significantly changed our results of expressed gene-levels. Moreover, we aimed to identify potential microRNA-biomarkers for subclinical hypothyroidism – markers that could be traced to offspring as well.

**Methods:**

From a cohort of at-term pregnant women undergoing planned cesarean section, 77 women had expression levels of the mitochondria-related genes Peroxisome Proliferator-activated Receptor-γ coactivator-1β (PGC-1β), mitochondrial Transcription Factor A (TFAM), Superoxide Dismutase 2 (SOD2) and Nuclear Respiratory Factor 2 (NRF-2) determined by qPCR from blood sampled in prior to delivery. Two TSH-cutoff levels defining subclinical hypothyroidism (> 3.0 and > 3.7 mIU/L) were applied for the procession of results, generating two data analyses of the same cohort. In 22 pairwise maternal-cord samples (subclinical hypothyroid/euthyroid-rate 0.5, TSH-cutoff > 3.0 mIU/L), microRNA-expressions (miRNA) were analyzed.

**Results:**

All gene expressions were lower in the subclinical hypothyroid group regardless of applied TSH-cutoff, but insignificant except for PGC-1β at TSH cutoff > 3.0 mIU/L. Two miRNAs (hsa-let-7d-3p and hsa-miR-345-5p) were upregulated in blood from women and offspring (cord blood) with subclinical hypothyroidism.

**Conclusions:**

A trend towards decreased mitochondrial gene expressions in subclinical hypothyroidism were demonstrated. The miRNAs hsa-let-7d-3p and hsa-miR-345-5p might be potential markers of maternal subclinical hypothyroidism. However, larger studies are needed to verify the findings.

**Supplementary Information:**

The online version contains supplementary material available at 10.1186/s13044-023-00180-6.

## Background

Subclinical hypothyroidism (SCH) is characterized by increased thyrotropin (TSH) and normal levels of the thyroid hormones thyroxine (T4) and triiodothyronine (T3). Thyroid hormones increase fat mobilization, stimulate insulin-dependent glucose uptake, gluconeogenesis and glycogenolysis [1]. By genomic and non-genomic actions, thyroid hormones regulate important cellular and metabolic processes, including mitochondrial function [2, 3]. On the genomic (nuclear) level, thyroid hormones regulate thyroid hormone nuclear receptors and thyroid receptor transcription activity [4]. These effects can be measured within hours to days [2]. On the non-genomic level, thyroid hormones more directly regulate mitochondrial energetics, mitochondrial biogenesis, induction of mitochondrial DNA and non-genomic actions in mitochondria [4]. These effects can be measured within seconds to minutes [2, 3]. Mitochondrial function can be evaluated by all these effects and not only by measuring energetics, which traditionally has been the most common approach. However, thyroid hormone regulation of mitochondria holds many unanswered and complex questions, and thus a detailed description of regulation mechanisms is yet to be detected [3].

Examples of thyroid regulated mitochondria-related transcripts (genomic level) are the co-activators from the PGC-1 family [5–8], the transcription factors Nuclear Respiratory Factor 1 (NRF-1) or 2 (NRF-2) [5, 9], mitochondrial Transcription Factor A (TFAM) [5, 6] and the antioxidant enzymes Superoxide Dismutase 1–3 (SOD1-3) [10, 11]. Expression of these mitochondria-related genes are all linked to mitochondrial biogenesis and function, and therefore, thyroid dysfunction may be associated with changes in these. Even subtle thyroid changes within normal reference values may lead to mitochondrial changes [12]. For instance, Kristensen et al. demonstrated that hemi-thyroidectomy significantly increased TSH (within normal range), and concomitantly influenced mitochondrial energetics in terms of increasing the mitochondrial membrane potential in mononuclear blood cells [13]. Also, the mitochondria-related genes peroxisome proliferator-activated receptor-γ coactivator-1α (PGC-1α) and superoxide dismutase 2 (SOD2) were downregulated [14]. Findings like this indicate a necessity to gain more knowledge of subclinical hypothyroid state and the association with cellular metabolism.

The number of mitochondria vary, dependent on tissue sort and energy demands [10, 15]. Likewise, thyroid hormone levels are regulated in different tissues by the expressions of different deiodinases. In placental and fetal tissue, the type 3 deiodinase (D3) is predominantly expressed and inactivates thyroid hormones, while type 2 deiodinase (D2) is also expressed in the placenta, where it deiodinates T4 to the active hormone T3 [1]. These mechanisms make pregnancy and thyroid function very interesting, as thyroid hormone exposure to the fetus seems very adequately tuned. As thyroid hormones regulate mitochondria, it is therefore worth taking a glance towards cellular processes concerning the “pregnancy load” on the thyroid function, as thyroid”normality” in pregnancy has been difficult to define and to understand the impact of.

In short, recommendations for the definition of subclinical hypothyroidism in pregnancy have in recent years been eagerly debated and changed from in 2011 (by the American Thyroid Association) and in 2012 (by the Endocrine Society) a definition in the third trimester of TSH > 3.0 mIU/L [16, 17], over TSH > 3.5 mIU/L in 2014 (by the European Thyroid Association) [18], to in 2017 local established references, with a maximum of 4.0 mIU/L (by the American Thyroid Association) [19]. Moreover, the clinical importance and the need for treatment of subclinical hypothyroidism have been discussed [20]. Studies are difficult to compare, as TSH levels are dependent on factors such as ethnicity, iodine supply, age, laboratory methods [21] and thyroid autoimmunity [22] as well as goitrogens, for instance from smoking [23].

In Denmark, the Danish Society of Endocrinologists in 2018 recommended to use TSH > 3.7mIU/L as the upper cutoff for the definition of subclinical hypothyroidism in the third trimester, in the absence of local laboratory pregnancy references [24]. However, this threshold was recently changed to TSH > 3.5 mIU/L [25] which underlines that definitions and clinicians´ perception are persistently changing, also within populations. In relation to the controversial debate concerning the clinical significance of sub-hypothyroid state in pregnancy, it seems increasingly important to gain more knowledge about thyroid impact, also on the cellular level.

To our knowledge, few studies have investigated mitochondrial changes in subclinical thyroid disorder in pregnancy [12]. As subclinical hypothyroidism may be rather common in pregnancy [12, 26], it seems relevant to assess thyroid regulated mitochondria-related gene expressions in subclinical hypothyroid pregnant women. Optimizing our understanding of metabolic effects, and thereby thyroid normality in pregnancy, could contribute to a more reliable definition.

Other regulators of mitochondria-related genes and of thyroid function are microRNAs (miRNA). A miRNA is a 19–25 nucleotides long non-coding RNA-strand that regulate gene expression at the posttranscriptional level, by binding to messenger RNA (mRNA) [27]. In this way, they either suppress mRNA translation or induce mRNA degradation [28]. Each miRNA can target several mRNAs, as one mRNA can be the target of several miRNAs [27, 28], and in this way miRNAs regulate both normal and pathological processes [29] in cell proliferation, differentiation, apoptosis and in metabolism [30]. Concerning the thyroid, miRNA research has primarily been centered around cancer. However, evidence exists that miRNAs are essential for thyroid gland development, thyroid hormone production, and for the regulation of locally expressed deiodinases in different tissues which adjust the bioavailability of thyroid hormones, and thereby their effects [31]. Knowledge of miRNA-expressions related to subclinical hypothyroidism is sparse.

Our hypothesis for this study was that a mitochondrial dysfunction could be present in subclinical hypothyroid pregnant women, reflected by a downregulated level of mitochondria related gene transcripts. Moreover, that a potential mitochondrial dysfunction could be traced in cord blood at delivery by biomarkers (miRNA) that would be present in the mothers, too.

### Aims

Aims of the present study were to

i) Compare mitochondrial biogenesis in euthyroid and subclinical hypothyroid pregnant women by assessing levels of TFAM, PGC-1β, NRF-2 and SOD2.
ii) Investigate if changing TSH-cutoff markedly affects results of gene-expression levels of TFAM, PGC-1β, NRF-1 and SOD2 in euthyroid vs. subclinical hypothyroid pregnant women.
iii) Compare mean levels of expressed miRNAs in euthyroid and subclinical hypothyroid pregnant women, and their offspring, and assess a potential relation to mitochondrial biogenesis.

## Methods

### Study population

**Fig. 1:**
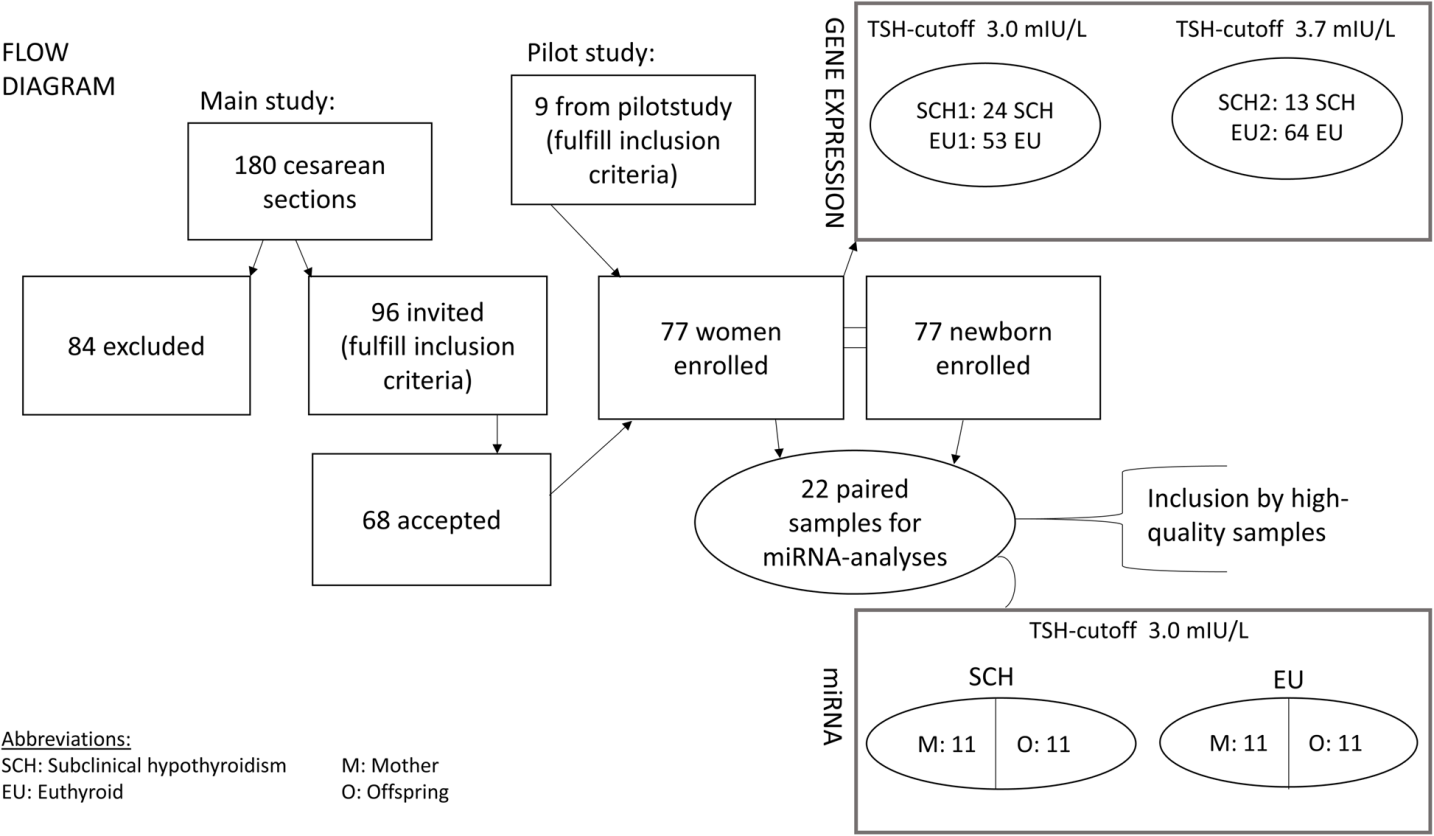
Flow diagram

In the present study (Fig. 1), medical journals for all planned cesarean sections were in advance reviewed to define exclusion and inclusion criteria. Afterwards, third trimester pregnant women were recruited by an interview and blood samples prior to a planned cesarean section. After delivery, their offspring were enrolled by cord blood samples. The women were included at Obstetrics Dept., Naestved Hospital, Denmark from January – February 2014 (pilot study) and again from June 2014—July 2015 (main study period).

Inclusion criteria: maternal age of at least eighteen years, an expected healthy, singleton pregnancy verified by a routine ultrasound scan in the second trimester and maternal health without any diseases or medication causing adverse effects to the fetus.

Exclusion criteria: multiple pregnancies, medical treated thyroid disease, diabetes (including gestational), preeclampsia or any other disease suspected to cause adverse effect to the fetus. Lack of informed verbal and written consent from both parents led to exclusion, too.

In the study period, a total of 180 planned cesarean sections were performed. Sampling logistics and laboratory capacity (*n* = 31), challenged family situation and missing full parental consent (*n* = 14) as well as positive exclusion criteria (*n* = 39) were the cause that only 96 women were invited to participate. Of these, 68 accepted (70.83%). In the pilot study, 9 were included, obtaining a total of 77 participating women. According to guidelines at inclusion time (TSH > 3.0 mIU/L) [16], 24 women fulfilled the criteria for SCH, and 13 according to recent Danish guidelines (TSH > 3.7 mIU/L) [19, 24]. The rest were euthyroid. According to the different guidelines, study results were analyzed as cohorts SCH1 (subclinical hypothyroid group 1) and EU1 (euthyroid group 1) for TSH cutoff 3.0 mIU/L and SCH2 (subclinical hypothyroid group 2) and EU2 (euthyroid group 2) for TSH cutoff 3.7 mIU/L.

### Maternal and cord metabolic analyses

A 3.5 mL heparinized blood sample was collected for same-day analysis of thyroid status, glucose, cholesterol and triglycerides. A serum gel tube (3.5 mL) was collected for thyroid peroxidase antibodies (anti-TPO) measurements.

Thyrotropin (TSH), free triiodthyronin (fT3) and free thyroxine (fT4) were analyzed by Siemens Dimension Vista System by an electrochemical luminescent immunoassay based on LOCI-technology (2008 Siemens Healthcare Diagnostics). Third trimester normal values for maternal TSH was defined as TSH: 0.3—3.0 mIU/L (EU1) or 0.3—3.7 mIU/L (EU2). The lowest value followed the standard used at the Dept. of Biochemistry at Naestved Hospital, and the upper value was in accordance with the different guidelines [16, 24]. Maternal normal values for fT3 and fT4 followed the references used at Naestved Hospital: fT4 = 8.5 – 26.0 pmol/L and fT3 = 2.7—6.1 pmol/L. Anti-TPO was measured on Kryptor by TRACE-technology (2005 Brahms Kryptor) with a detection limit of 11 kU/l. An anti-TPO > 60 kU/l was considered a positive test. To compare metabolism, glucose, total-cholesterol, low-density lipoprotein cholesterol (LDL), high-density lipoprotein cholesterol (HDL) and triglycerides were analyzed by Siemens Dimension Vista System (2008 Siemens Healthcare Diagnostics) by photometry.

### Mitochondria-related RNA sequencing

For reverse transcription by quantitative Polymerase Chain Reaction (qPCR), a 3 mL Tempus™ Blood RNA Tube (Life Technologies, Denmark) containing a reagent that instantly stabilizes intracellular RNA, was used. Samples were subsequently frozen and stored at -80 °C until analysis. The laboratory technician was blinded to which thyroid groups the samples belonged. Sample collection and handling were performed according to the manufacturer´s recommendation.

RNA was purified using PerfectPure™ RNA Blood kit (5 Prime, AH Diagnostics) in accordance with the manufacturer’s recommendations. The integrity of RNA was characterized by the RNA integrity number (RIN) measured on an Agilent 2100 Bioanalyzer (Agilent Technologies, Copenhagen, Denmark). For analysis, a RIN value above 7.0 was considered acceptable. Whole blood gene expressions of PGC-1β, TFAM, NRF-2 and SOD2 were examined. Table 1 presents the primer sequences used.

**Table 1:** Primer sequences

DNA name	Forward primers (5′–3′)	Reverse primers (5′–3′)
PGC-1β	5’-CTC CTA CGG GGA CCC CAG AT-3’	5’-CCA CTG TCA AGG TCT GCT CA-3’
NRF-2	5’-GCC GCT TGG AGG CTC ATC TCA-3’	5’-GCA ATT CTG AGC AGC CAC TTT ATT CT-3’
SOD2	5’-AGG GGA GTT GCT GGA AGC CAT-3´	5’-CCC ACA CAT CAA TCC CCA GCA GT-3’
TFAM	5’ -AGC TCA TGG ACT TCT GCC AGC A-3’	5’-CCT GCC TCC ATA ATA TAA GGA AAC AAG AGT-3’
18S	5’-TAC CAC ATC CAA GGA AGG CAG CA-3’	5’-CTG CAG CAA CTT TAA TAT ACG CTA T-3’

Reverse transcription was performed using Quanti-Tect Reverse Transcription Kit (Qiagen, Copenhagen, Denmark). A SYBR Green based qRT-PCR of PGC-1β, TFAM, NRF-2 and SOD2 mRNA was measured on a LightCycler 480 (Roche). To correct for potential variation in cDNA loading and quantity, the measured gene transcripts levels were normalized to the expression of ribosomal 18 S RNA. The qRT-PCR reactions were performed according to the LightCycler standard protocol. The qPCR reaction mixture included 1 × LightCycler FastStart DNA Master PLUS SYBR Green I (Roche, Copenhagen, Denmark) and 1 μl mRNA specific- and 0.5 μl of the 18 S primers, 1 μmol/l respectively. For each reaction 5 μl template cDNA and sterile water were added in a total reaction volume of 20 μl. Cycling conditions were 95 °C for 10 min, followed by 45 cycles at 95 °C for 10 s, and 60 °C for 1 min. All quantitative RT-PCR measurements were performed in triplicates. Melting curves were completed for the control of unspecific DNA amplification after each run. Unit of measurement for gene expressions was arbitrary units (a.u.).

Some results did not reach acceptable quality, and were excluded. The excluded samples were from 2 women with TSH > 4 mIU/L, and from 2 with TSH < 2 mIU/L, BMI-range 25.1–27.6. Therefore, only 73 samples presented full gene profile results (EU1 *n* = 51, SCH1 *n* = 22, EU2 *n* = 62, SCH2 *n* = 11), and for TFAM one result could be added to euthyroid-groups (EU1 *n* = 52, EU2 *n* = 63).

The amount obtainable of cord blood were too low for qPCR analysis. This was due to a very sparse amount of blood left for the Tempus tube after collection of blood for standard pH-measurements (2–4 mL), for thyroid function-tests (3.5 mL), anti-TPO measurements (3.5 mL), for flow cytometry (4 mL) (results published in [26, 32]) and for miRNA analyses (4 mL). Seldom, blood was left in the cord after collection for these other tubes. If more blood was left, it was very difficult to do more sampling due to coagulation.

The choice of mitochondria-related genes were based on the fact that these different genes reflect mitochondrial function differently [5, 11, 33], and due to former laboratory experience [6, 34].

### Maternal and cord micro-RNA panel

For miRNA-screening, only a minor number of participants were chosen due to cost of analyses. Twenty-two maternal samples (11 euthyroid, 11 subclinical hypothyroid) and the associated cord samples were chosen randomly, as they fulfilled quality criteria concerning sufficient blood volume and no signs of hemolysis by the manufacturer´s primary quality check.

One EDTA-plasma fraction was sampled for miRNA-analysis and subsequently frozen and stored at -80 °C until shipping for analysis.

The real-time PCR panel analysis was performed by Exiqon the following way: Each RNA sample was successfully reverse transcribed (RT) into cDNA and run on the miRCURY LNA™ Universal RT microRNA PCR Human Panel I + II (752 assays). For normalization of data, the average of the assays detected in all samples was applied as it was the most stable normalizer. Numbers of miRNA present were detected and the quantification cycle (Cq) value of the global mean for each of the samples was identified.

Each individual amplification product on PCR panels was scrutinized by melting curve analysis, calculation of amplification efficiency and comparison of Cq value to background level in a negative control sample. For quality control, two types of RNA-spike-ins kits (Exiqon) were used: For RNA isolation control, UniSp2, UniSp4, UniSp5 (Exiqon) were added to the purification to detect any differences in extraction efficiency. For cDNA synthesis control, UniSp6 (Exiqon) was added in the RT reaction, giving the opportunity to evaluate it. In addition, a DNA spike-in (UniSp3, Exiqon) was present on all panels, to indicate inhibitions at the qPCR level by deviations in this reaction.

Exiqon was blinded to which thyroid groups the samples belonged.

### Statistics

Two-tailed t-test was used to compare data with a normal distribution. Otherwise, results were handled non-parametrically (non-normality confirmed by Shapiro-Wilks test). Wilcoxon Rank-sum test was used to compare level of differences. Spearman´s rho ρ was used to access correlations. A *p*-value < 0.050 was considered statistically significant. Data processes were carried out using STATA version 15.0 for Windows (StataCorp LP, College station, TX, USA).

Regarding miRNA, Exiqon analysed the data as follows: A Principal Component Analysis (PCA) was used to reduce the dimension of the large data set and identify the miRNAs with the largest variation across samples. The most differentially expressed miRNAs were analysed by t-test and a Benjamini–Hochberg correction. The analyses were made in accordance with the definition of SCH at that time (TSH > 3.0 mIU/L).

### Power analysis

A calculation with 90% strength at the 5% significance level (alpha) specified recruitment of 242 euthyroid and 48 subclinical hypothyroid women to show a significant decrease in subclinical hypothyroid group TFAM. Calculations were based on a mean value of 1.70 in euthyroid and 1.19 in subclinical hypothyroid group, and a common SD of 1.00. For PGC-1β, NFR-2 and SOD2, recruitment of 252 euthyroid and 50 subclinical hypothyroid women was necessary to demonstrate a significant difference. Calculations were based on a mean value of 4.70 in the euthyroid and 3.80 in the subclinical hypothyroid group, and a common SD of 2.00. Calculations were based on a rate of subclinical hypothyroidism /euthyroidism = 0.20 (TSH cutoff 3.7 mU/L).

Calculations were performed retrospectively, as this study was performed in supplement to a flow cytometric evaluation of mitochondrial function (primary outcome) of the same cohort (unpublished results [26]), preceded by a method study [32]. Unfortunately, it was not possible to obtain the calculated power of the study, due to health circumstances in the research team, which interrupted the main study prematurely.

## Results

### Maternal metabolic profiles

Table 2 depicts relevant maternal data. Age, BMI and gestational age were comparable between cohorts. In general, other metabolic parameters such as blood glucose level, total cholesterol, LDL, HDL and triglycerides did not differ (Table 2). Thus, other metabolic differences that could affect our results on the mitochondrial level were not present.

**Table 2:** Maternal metabolic profile

	**Euthyroid 1** *N* = 53	**Subclinical hypothyroid 1** *N* = 24	***p*-value**	**Euthyroid 2** *N* = 64	**Subclinical hypothyroid 2** *N* = 13	***p*-value**
**Cutoff**	TSH < = 3.0	TSH > 3.0		TSH < = 3.7	TSH > 3.7	
**Age**, years, SD	32.36, SD 5.14	32.54, SD 5.27	0.886^A^	32.20, SD 5.20	33.46, SD 4.91	0.425^A^
Range	(25–46)	(21–43)		(21–46)	(25–39)	
**Gestational age by delivery**						
Median /week + days	39 + 1	39 + 1	-	39 + 1	39 + 2	-
Range / week + days	37 + 1–41 + 4	36 + 6 – 40 + 1		36 + 6—41 + 4	38 + 5 – 39 + 6	
**BMI, kg/m**^**2**^** pre-pregnancy**						
Median	24.1	22.6	0.117	24.1	22.3	0.554
Range	16.4–50.8	16.9–43.9		16.4–50.8	16.9–43.9	
**BMI, kg/m**^**2**^** at delivery**						
Median	29.8	28.1	0.096	29.0	28.6	0.822
Range	21.3–51.1	22.3–47.5		21.3–51.1	23.7–47.5	
**Smoking**						
Number (%)	3 (5.7%)	3 (12.5%)	-	5 (7.8%)	1 (7.7%)	-
**Glucose, mmol/l**						
Median	4.3	4.3	0.307	4.3	4.2	0.413
Range	(3.6–7.2)	(3.7–5.2)		(3.6–7.2)	(3.8–4.6)	
**TSH, mIU/L**						
Median	1.9	3.8	< 0.001	2.0	4.3	< 0.001
Range	(0.3–3.0)	(3.1–5.6)		(0.3–3.5)	(3.8–5.6)	
**fT3, pmol/L**						
Median	3.8	3.8	0.716	3.8	4.1	0.121
Range	(2.7–4.7)	(3.1–6.4)		(2.7–4.8)	(3.4–6.4)	
**fT4, pmol/L**						
Median	11.8	11.3	0.341	11.8	11.0	0.373
Range	(8.5–15.9)	(8.7–14.6)		(8.5–15.9)	(8.7–14.6)	
**Anti-TPO > 60, kU/l**						
Number (%)	2 (3.8%)	1 (4.2%)	-	2 (3.1%)	1 (7.7%)	-
**Total-Cholesterol, mmol/l**						
Median	6.5	6.4	0.873	6.4	6.6	0.458
Range	(4.1–10.5)	(4.8–10.0)		(4.1–10.5)	(5.1–9.3)	
**LDL, mmol/l**						
Median	3.2	3.3	0.699	3.2	3.4	0.557
Range	(1.0–6.9)^a^	(1.6–6.4)^b^		(1.0–6.9)^c^	(2.5–5.4)^d^	
**HDL, mmol/l**						
Median	1.7	1.9	0.128	1.7	2.0	0.058
Range	(0.9–3.3)	(1.1–2.5)		(0.9–3.3)	(1.1–2.5)	
**Triglycerides, mmol/l**						
Median	2.9	2.5	0.063	2.9	2.4	0.226
Range	(1.6–6.7)	(1.2–6.4)		(1.2–6.7)	(1.8–6.4)	

Values presented as numbers and percentages, means, standard deviations (SD) and range or median and range

^A^Two-tailed t-test

^a^*N* = 46

^b^*N* = 22

^c^*N* = 56

^d^*N* = 12

### Mitochondria-related gene expression in relation to thyroid status

**Fig. 2:**
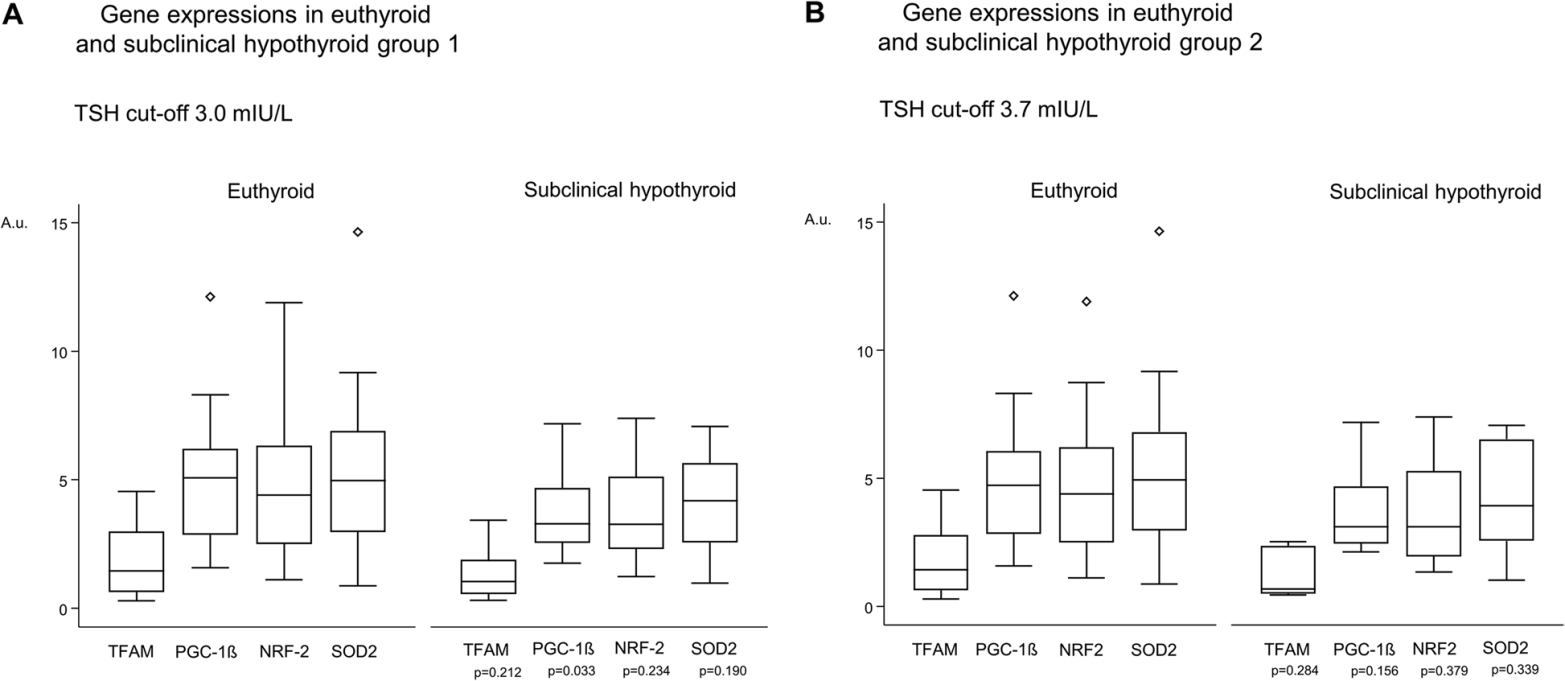
**A**-**B** Maternal euthyroid and subclinical hypothyroid gene-expressions. **A** by TSH-cutoff 3.0 mIU/L and **B** by TSH-cutoff 3.7 mIU/L. X-axis: TFAM, PGC1-β, NRF-2 and SOD2 expressions. Y-axis: Gene expression target/reference / a.u. Boxes = lower and upper quartile, horizontal line in box = median value, whiskers = range

Mitochondria-related gene expression levels in euthyroid and subclinical hypothyroid pregnant women are depicted by the different reference standards of TSH in Fig. 2A (TSH cutoff 3.0 mIU/L) and Fig. 2B (TSH cutoff 3.7 mIU/L).

Irrespective of TSH cutoff, every gene showed a lower expression level in pregnant women with subclinical hypothyroidism compared with euthyroids. However, apart from PGC-1β expression in group EU1 vs. SCH1 (*p* = 0.033), results were non-significant (Fig. 2A-B). Notably, the mitochondria-related genes were positively correlated with each other (Table 3).

**Table 3:** Spearman´s correlation coefficients (ρ) of mitochondria-related gene expressions

Gene correlates	EU1 / ρ (*p*-value)	SCH1 / ρ (*p*-value)	EU2 / ρ (*p*-value)	SCH2 / ρ (*p*-value)
PGC-1β – TFAM	0.861 (*p* < 0.001)	0.885 (*p* < 0.001)	0.875 (*p* < 0.001)	0.782 (*p* = 0.005)
PGC-1β—NRF-2	0.849 (*p* < 0.001)	0.897 (*p* < 0.001)	0.859 (*p* < 0.001)	0.836 (*p* = 0.001)
PGC-1β—SOD2	0.778 (*p* < 0.001)	0.839 (*p* < 0.001)	0.796 (*p* < 0.001)	0.873 (*p* = 0.001)
TFAM – NRF-2	0.961 (*p* < 0.001)	0.974 (*p* < 0.001)	0.964 (*p* < 0.001)	0.955 (*p* < 0.001)
TFAM – SOD2	0.819 (*p* < 0.001)	0.883 (*p* < 0.001)	0.823 (*p* < 0.001)	0.909 (*p* < 0.001)
NRF-2 – SOD2	0.906 (*p* < 0.001)	0.938 (*p* < 0.001)	0.908 (*p* < 0.001)	0.964 (*p* < 0.001)

In supplementary section, maternal TSH is plotted against the different gene expressions (Fig. S1).

### MicroRNA profiles

**Fig. 3:**
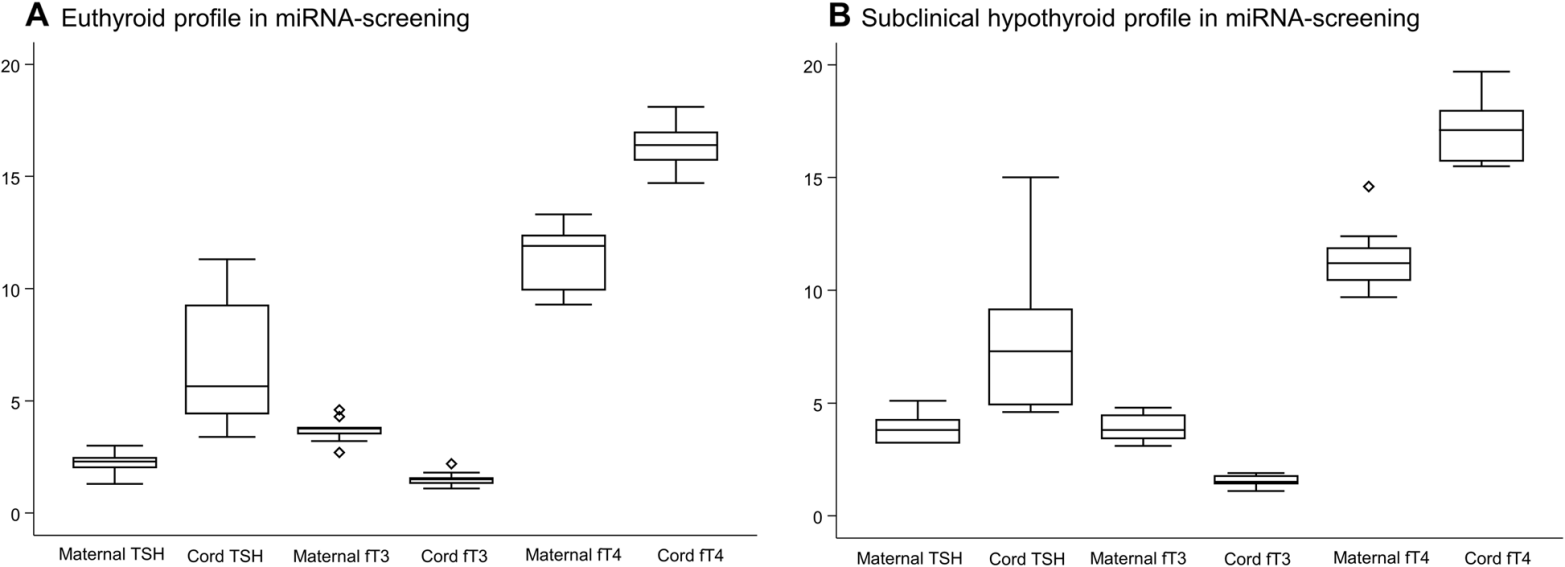
**A**-**B** Maternal and offspring (cord) thyroid status in euthyroid and subclinical hypothyroid cohort, in miRNA screening. X-axis: maternal and cord TSH, fT3 and fT4. Y-axis: Units: TSH-values / mIU/L, fT3-and fT4-values / pmol/L. Boxes = lower and upper quartile, horizontal line in box = median value, whiskers = range

In the miRNA-study, only the TSH-cutoff of 3.0 mIU/L was used to define euthyroidism vs. subclinical hypothyroidism, as the analyses were made by Exiqon according to the definition of subclinical hypothyroidism at that time [16]. Apart from maternal TSH (p < 0.001), thyroid profiles did not differ between euthyroid and subclinical hypothyroid women or offspring (Fig. 3A-B).

Mean maternal age did not differ significantly, and in both cohorts, the women delivered at mean gestational age of 39 + 1 (Table 4). The women with subclinical hypothyroidism were leaner, but not significant. Metabolic profile differed a bit from the profile in the genomic study, but there were no significant differences in blood glucose or lipids of euthyroid and subclinical hypothyroid women (Table 4).

**Table 4:** Profile of euthyroid (EU) and subclinical hypothyroid (SCH) group in miRNA-analysis

	EU *n* = 11	SCH *n* = 11	*p*-value (rank-sum)
**Cutoff**	TSH < = 3.0	TSH > 3.0	
**Maternal age, years,**			
Mean, SD	32.5, SD 3.7	33.5, SD 4.3	0.564^A^
Range	(27–39)	(27–39)	
**Gestational-age, week + days,**			
Median	39 + 1	39 + 1	-
Range	(37 + 6–41 + 4)	(38 + 1–39 + 8)	
**BMI, kg/m**^**2**^** pre-pregnancy**			
Median	28.20	23.20	0.793
Range	(16.4–50.8)	(19.5–36.3)	
**BMI, kg/m**^**2**^** at delivery**			
Median	34.10	28.70	0.309
Range	(21.3–51.1)	(23.6–40.1)	
**Smoking**			
Number (%)	0 (0.00%)	2 (18.18%)	-
**Child gender**			
Female/male, number	6/5	5/6	-
Female/male, %	54.55/45.45	45.45/54.55	
**Child birthweight, gram**			
Mean, SD	3518, SD 510	3609, SD 439	0.659^A^
Range	(2750–4620)	(2950–4202)	
**Glucose, mmol/l**			
Median	4.40	4.30	0.354
Range	(3.7–5.1)	(3.9–4.6)	
**Total-Cholesterol, mmol/l**			
Median	5.30	6.20	0.139
Range	(4.3–9.8)	(5.1–8.8)	
**LDL, mmol/l**			
Median	2.40	2.90^a^	0.057
Range	(1.1–6.9)	(2.3–4.8)	
**HDL, mmol/l**			
Median	1.60	1.70	0.947
Range	(1.2–3.3)	(1.1–2.5)	
**Triglycerides, mmol/l**			
Median	2.80	2.60	0.532
Range	(1.9–4.3)	(1.2–6.4)	

Values presented as means, standard deviations (SD) and range, except when number and percentage are listed

^A^Two-tailed t-test

^a^*N* = 9

Total cholesterol in cords varied from < 1.3 to 1.8 mmol/l, LDL from unmeasurable to 0.9 mmol/l, HDL from 0.4 to 0.8 mmol/l and triglycerids from < 0.1 to 0.3 mmol/l with no difference between groups (data not presented). Birthweights were very alike in the two groups, and rate of gender in the two groups were not significantly different (Table 4).

In all of the samples, 219 identical miRNAs could be presented. In each sample, an average of 407 miRNAs was detectable. When comparing maternal subclinical hypothyroid and euthyroid group, 30 miRNAs were differentially expressed by t-test, but none significantly after performing the Benjamini-Hochberg (BH) correction. For cord samples, 29 miRNAs were differentially expressed by t-test, and one persisted to be significant after the BH-correction. The 20 most differentially expressed miRNAs in maternal respective cord samples are depicted in Tables 5 and 6.

**Table 5:** Most differentially expressed miRNA´s between subclinical hypothyroid (SCH1) and euthyroid (EU1) maternal blood samples

*miRNA*	*Mean dCq (SD)* *EU1*	*Mean dCq (SD)* *SCH1*	*Fold change*	*P-value* *t-test / BH adj*	*Target gene*
hsa-miR-96-5p	-4.0 (0.46)	-4.9 (0.61)	-1.9	0.001 / 0.380	
hsa-miR-223-3p	8.9 (0.19)	9.3 (0.32)	1.3	0.003 / 0.380	
hsa-miR-7–1-3p	-2.9 (0.63)	-2.1 (0.35)	1.7	0.004 / 0.380	
hsa-miR-197-3p	1.2 (0.17)	1.5 (0.29)	1.3	0.004 / 0.380	
hsa-miR-24–2-5p	-1.7 (0.31)	-1.3 (0.34)	1.4	0.005 / 0.380	
hsa-miR-23a-3p	5.9 (0.14)	6.1(0.20)	1.2	0.005 / 0.380	
hsa-miR-1468-5p	-6.5 (0.37)	-7.9 (0.93)	-2.6	0.007 / 0.450	
**hsa-miR-345-5p**	0.03 (0.33)	0.51 (0.42)	1.4	0.007 / 0.450	
hsa-miR-664a-3p	-3.7 (0.38)	-3.2 (0.40)	1.4	0.008 / 0.450	
hsa-miR-502-3p	-2.3 (0.44)	-3.0 (0.57)	-1.5	0.010 / 0.450	NRF-1, SOD2-3, TFAM
hsa-miR-486-5p	3.5 (0.41)	3.0 (0.43)	-1.4	0.010 / 0.450	NRF-1, SOD2-3
hsa-miR-125a-5p	1.1 (0.40)	1.6 (0.52)	1.5	0.013 / 0.490	NRF-1, TFAM
hsa-miR-223-5p	-2.3 (0.42)	-1.8 (0.55)	1.5	0.013 / 0.490	
hsa-miR-193a-5p	-5.7 (0.70)	-4.5 (1.2)	2.3	0.014 / 0.490	NRF-1, SOD1-3, TFAM
hsa-miR-551a	-5.1 (0.72)	-3.9 (1.2)	2.3	0.015 /0.490	No relevant
hsa-miR-146b-5p	-1.6 (0.31)	-2.2 (0.61)	-1.5	0.018 / 0.540	SOD3, TFAM
hsa-miR-188-5p	-3.9 (0.62)	-4.6 (0.68)	-1.6	0.020 / 0.560	SOD1 + 3, TFAM
**hsa-let-7d-3p**	2.1 (0.18)	2.3 (0.21)	1.2	0.021 / 0.560	
hsa-miR-99b-5p	-0.44 (0.45)	0.012 (0.42)	1.4	0.024 / 0.600	
hsa-miR-363-3p	0.51 (0.32)	0.15 (0.37)	-1.3	0.026 / 0.600

**Table 6:** Most differentially expressed miRNA´s between subclinical hypothyroid (SCH1) and euthyroid (EU1) cord blood samples

*miRNA*	*Mean dCq (SD)* *EU1*	*Mean dCq (SD)* *SCH1*	*Fold change*	*P-value* *t-test / BH adj*	*Target gene*
hsa-miR-340-3p	-4.8 (0.54)	-6.6 (0.57)	-3.4	< 0.001 / 0.015	
hsa-miR-331-3p	-0.90 (0.22)	-1.3 (0.30)	-1.3	0.002 / 0.460	NRF-1, SOD1-3, TFAM
hsa-miR-597-5p	-9.8 (0.34)	-8.4 (0.52)	2.8	0.003 / 0.460	
hsa-miR-363-5p	-6.8 (0.39)	-5.6 (0.37)	2.4	0.004 / 0.460	
hsa-miR-654-5p	-2.5 (0.82)	-3.5 (0.47)	-2.0	0.008 / 0.700	NRF-1, SOD1-2, TFAM
hsa-miR-642a-5p	-7.3 (1.3)	-5.5 (0.99)	3.3	0.010 / 0.700	
**hsa-let-7d-3p**	1.7 (0.28)	2.1 (0.34)	1.3	0.011 / 0.700	
hsa-miR-208a-3p	-7.1 (0.52)	-6.0 (0.33)	2.1	0.012 / 0.700	
hsa-miR-532-3p	-1.2 (0.31)	-0.73 (0.41)	1.3	0.013 / 0.700	SOD2-3, TFAM
hsa-let-7 g-3p	-3.0 (0.50)	-2.5 (0.38)	1.4	0.016 / 0.700	
hsa-miR-107	2.8 (0.31)	2.4 (0.24)	-1.2	0.016 / 0.700	NRF-1, SOD1-3, TFAM
hsa-miR-424-3p	-2.2 (0.72)	-1.5 (0.37)	1.6	0.019 / 0.770	
hsa-miR-202-3p	-5.7 (0.63)	-4.7 (0.93)	2.0	0.023 / 0.770	
hsa-let-7b-3p	-3.2 (0.61)	-2.6 (0.35)	1.4	0.024 / 0.770	
hsa-miR-153-3p	-5.7 (0.61)	-6.8 (1.4)	-2.2	0.024 / 0.770	
hsa-miR-501-3p	-2.2 (0.68)	-1.6 (0.34)	1.5	0.026 / 0.770	NRF-1, SOD3, TFAM
**hsa-miR-345-5p**	1.4 (0.36)	1.8 (0.45)	1.3	0.028 / 0.770	
hsa-miR-518d-5p	-5.0 (1.1)	-6.2 (1.2)	-2.3	0.028 / 0.770	NRF-1, SOD3, TFAM
hsa-miR-99b-3p	-6.8 (1.4)	-5.4 (1.1)	2.6	0.034 / 0.800	
hsa-miR-26b-5p	-0.92 (0.53)	-1.5 (0.74)	-1.5	0.037 / 0.800	

In other words, we were able to demonstrate biomarker (miRNA) differences related to TSH level by t-test. However, after BH-adjustment, only one (hsa-miR-340-3p) remained significant. Interestingly, two miRNAs (hsa-let-7d-3p and hsa-miR-345-5p) were equally upregulated in subclinical hypothyroid maternal and cord blood, which suggest they may be markers of SCH.

From the microPIR database [35], information about relevant target genes for the different miRNA´s was obtained. These are presented in Tables 5 and 6.

## Discussion

### Mitochondria-related gene expression in relation to thyroid status

The results of mitochondria-related gene expressions in relation to thyroid status presented here were insignificant, except for PGC-1β expression at TSH-cutoff 3.0 mIU/L. The insignificant findings in this study are most likely caused by the too small number of participants, which increases the risk of type-II errors. However, as the decrease in absolute terms in gene expressions in subclinical hypothyroid cohort was persistent—irrespective of TSH-cutoff applied—we suggest that a larger study could be appropriate, to investigate these very interesting findings more thoroughly.

To support the theory that subclinical hypothyroidism is associated with decreased expression of mitochondria-related genes, other studies have demonstrated noteworthy changes by overt hypothyroidism. Sinha et al. demonstrated decreased expression of the mitochondria-related genes NRF-1 α, PGC-1α and TFAM in brain tissue of hypothyroid rodents [36], and Sagliocchi et al. demonstrated decreased SOD2 levels in hypothyroid muscles compared to control muscles, and oppositely increased SOD2 levels in hyperthyroid muscles [37]. Overall, these are in accordance with our findings.

Another noteworthy finding in the present study is the demonstration of positive correlations between the investigated mitochondria-related genes, confirming they are part of a common regulatory pathway. To our knowledge, only few studies have investigated the correlation between regulators of mitochondrial function [38], but Fabricius et al. had similar results with positive correlations between expressed mitochondria-related genes within specific tissue types [6]. They investigated expressions in smooth muscle tissue (uterus), white adipose tissue and blood of 17 persons and found different levels of expressions in different tissues. PGC-1β and TFAM were most pronouncedly expressed in smooth muscle tissue, while NRF-1 and NRF-2 were expressed lowest in adipose tissue. In blood, all genes had a low, but similar expression [6] which makes it ideal for comparative studies where the objective is to detect small changes in expression between groups. This, and the factor that blood is easy obtainable, have led us to use blood in the present study.

PGC-1β was chosen as it is a co-activator that respond rapidly and robust to thyroid hormones by binding to different transcription factors that induce mitochondrial biogenesis [8] and energy metabolism in terms of regulation of beta-oxidation of fatty acids and oxidative phosphorylation in mitochondria [7]. Moreover, it regulates targets of mitochondrial fusion and fission, which is fundamental for mitochondrial repair and cell fate [39]. Functionally, it appears related to PGC-1α but are probably more energy inducing as its activation leads to a higher level of coupled respiration [5].

NRF-2 is a DNA-binding transcription factor implicated in activating cytochrome oxidase expression, and controlling nucleus-encoded subunits of cytochrome oxidase [5]. Together, NRF-2 and NRF-1 influence the expression of nuclear genes related to mitochondrial function [5]. It is well known that NRF-1 is a target of regulation for every member of the PGC-1 family, but studies in mouse tissue and cultured cells suggest NRF-2 as a target, too [5]. For that reason, it seemed important to choose NRF-2 in the present study.

SOD2 is involved in intracellular response to oxidative stress. In most cells, it is present in abundant amounts where it neutralizes reactive oxygen species (ROS), by accepting unpaired electrons [10, 11]. SOD2 differs from the other Superoxide Dismutases by its localization *inside* the mitochondrial matrix [11].

TFAM is a mtDNA transcription and maintenance factor that is regulated by the PGC-1 family (including PGC-1β) [5]. TFAM organizes the mitochondrial genome and is implicated in cell survival [33].

By the choice of transcription factors that regulate different compartments related to mitochondria, and a co-activator that regulate at least one of these, we hoped to demonstrate a common trend of changes in response to increasing TSH, and in absolute terms we succeeded in this.

### MicroRNA profiles

In the present study, we demonstrated a general variety of expressed miRNAs in maternal vs. cord samples. These different expressions were most likely caused by the different composition of maternal vs. cord blood. Cord blood contain stem cells, and another metabolism is present in cord blood compared to adult blood [32]. This different metabolism was reflected by the very different levels of thyroid hormones and lipid status in cord blood.

We demonstrated a tendency of different miRNA-expressions related to thyroid status, and perhaps identified a potential biomarker for maternal subclinical hypothyroidism (hsa-miR-340-3p). Interestingly, two potential biomarkers for subclinical hypothyroid disease in the woman and in her offspring were identified (hsa-let-7d-3p and hsa-miR-345-5p), implicating a potential effect on the offspring to maternal subclinical hypothyroid disease. In a recent overview of miRNAs linked to autoimmune thyroid disease, none of these miRNAs were described [40]. On the contrary, one of those isoforms (hsa-let7d) has been described to be highly expressed by normal functioning thyroid glands [41], which is why this upregulation in subclinical hypothyroid state is very interesting. However, Massolt et al. [42] investigated the expression of a panel of 384 selected miRNAs in different thyroid states of 13 thyroidectomized patients in treatment due to differentiated thyroid cancer. Some had suppressed TSH, and some had a very high TSH. In contrast to our study, they did not find any differences in expression of miRNAs in relation to thyroid status [42]. This could be caused by their smaller number of study participants, or perhaps by choice of other markers. Larger studies of miRNAs as biomarkers for changed metabolism in relation to thyroid disease would be appropriate to gain more knowledge about this field.

We searched the microPIR [35], a database that contains information about promoter interactions. The miRNAs hsa-let-7d-3p and hsa-miR-345-5p were not identified in it. However, every miRNA with accessible information had the specific mitochondria-related genes, the current paper assesses, among its targets (or the “family members” NRF-1, SOD1, SOD3), except from hsa-miR-551a. We also searched the TargetScanHuman database [43] and the miRBase [44] for a relation between the most noteworthy miRNAs in this study (hsa-let-7d-3p, hsa-miR-345-5p, hsa-miR-340-3p) and interactions with the examinated gene expressions in this study, but with no positive results.

In future studies, it could be beneficial to use miRNA mimics or inhibitors in cellular models to test if the identified miRNAs are directly associated with subclinical hypothyroidism, or if their effects are merely through regulation of gene targets already known.

In the microPIR database [35], PGC-1β was not listed as a target of regulation by miRNAs. Instead, PGC-1β is known to be a coactivator and a regulator of miRNAs [5]. This is in accordance with a study of thyroid tumor cells, with a high content of functional mitochondria, where evidence was found that coactivators of the PGC-1 family control mitochondrial function by regulation of miRNA expression [38].

Strengths to the study were comparable study groups in terms of gestational age and delivery method as well as metabolic profiles. Moreover, in the miRNA-study, the ratio of offspring gender was acceptable, factors that otherwise may influence miRNA expression [45].

Weaknesses were primarily lack of power, but also supplementary analyzes of cord blood quality could have been performed locally [46]. However, Exiqon is experienced in miRNA services, and therefore the plasma quality tests were run by their standards. Regarding collection and freezing, the samples were handled in a standardized way. However, variation in time delay from delivery to possibility of sampling after the cord clamping, could not be avoided, even though the standardization should minimize this aspect.

Minor weaknesses were the increased rate of smoking in the subclinically hypothyroid group compared to the euthyroid group in the miRNA-study, as smoking might modulate expressions. However, smoking in pregnancy is associated with a lower TSH than in non-smokers [47], which we have also demonstrated in our material [26].

To evaluate the overall interaction between maternal and fetal environment, it would have been beneficial (had the study been larger) to add measurements of gene- and miRNA expressions in placental tissue, and gene expressions in cord blood, too.

## Conclusions

Although non-significant, this study demonstrates a decrease in absolute terms in expression-level of the mitochondria-related genes PGC-1β, TFAM, NRF-2 and SOD2 in third trimester pregnant women with subclinical hypothyroidism. Two potential miRNA markers of subclinical hypothyroidism in maternal and cord blood (offspring) and one potential maternal miRNA marker were identified, and a variety of miRNAs with mitochondria-regulating genes as targets had different expressions in subclinical hypothyroid vs. euthyroid maternal samples. This could indicate changes on the cellular and metabolic level by increased TSH, even though the measured levels of thyroid hormones are considered normal in subclinical hypothyroidism. Therefore, this study may facilitate new research on thyroid regulation of mitochondria on the cellular level and in relation to heritage, and we encourage further research due to the small sample size in our study. Expression of metabolic biomarkers could be helpful in establishing the correct cutoff for subclinical hypothyroid disease in pregnancy, as they could help to understand normal and pathological processes and thereby determine the biological normal cutoff. In this way, clinical decision making of “to treat or not to treat” a patient could be supported.

### Supplementary Information

**Additional file 1.**

## Data Availability

The data that support the findings of this study are available from the main author, but restrictions apply to the availability of these data, which were used under license for the current study, and so are not publicly available. Data are however available from the authors upon reasonable request and with permission of the Danish Data Protection Agency. The Exicon Project Report, describing miRNA data analysis, is also available on request.

## Abbreviations

TSH: Thyrotropin
SCH: Subclinical hypothyroidism
qPCR: Quantitative Polymerase Chain Reaction
miRNA: Micro RNA
PGC-1β: Peroxisome Proliferator-activated Receptor-γ coactivator-1β
TFAM: Mitochondrial Transcription Factor A
SOD2: Superoxide Dismutase 2
NRF-2: Nuclear Respiratory Factor 2
