# Supplementary figures and images for: Expressions of mitochondria-related genes in pregnant women with subclinical hypothyroidism, and expressions of miRNAs in maternal and cord blood
Source: Thyroid Res. 2023 Sep 18;16:38. doi: 10.1186/s13044-023-00180-6 (PMC10506244; doi:10.1186/s13044-023-00180-6)

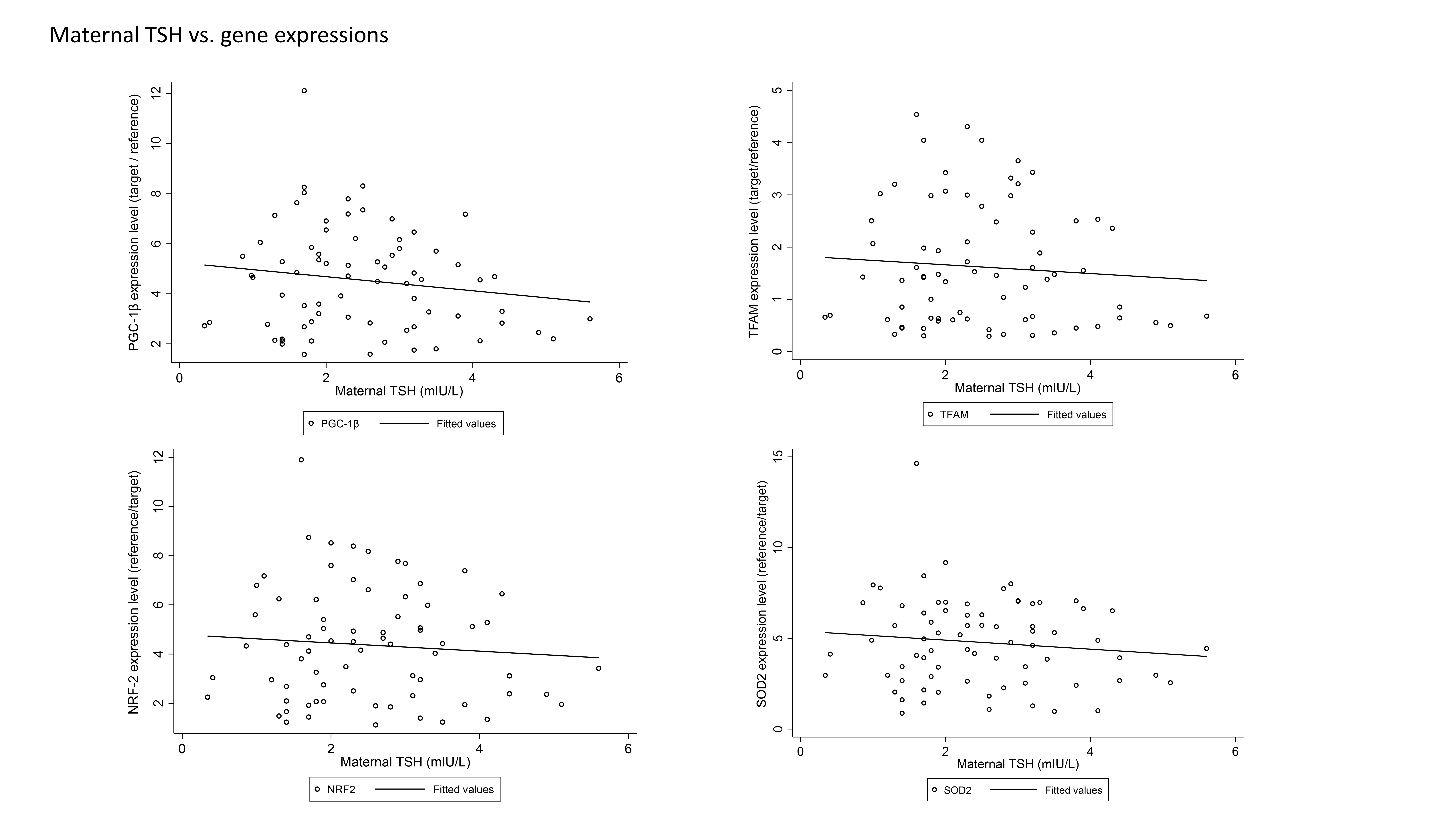

Supplement: Supplementary file 1 — Additional file 1. [file 13044_2023_180_MOESM1_ESM.tif]
